# An artificial intelligence-based nerve recognition model is useful as surgical support technology and as an educational tool in laparoscopic and robot-assisted rectal cancer surgery

**DOI:** 10.1007/s00464-024-10939-z

**Published:** 2024-07-29

**Authors:** Kazuya Kinoshita, Tetsuro Maruyama, Nao Kobayashi, Shunsuke Imanishi, Michihiro Maruyama, Gaku Ohira, Satoshi Endo, Toru Tochigi, Mayuko Kinoshita, Yudai Fukui, Yuta Kumazu, Junji Kita, Hisashi Shinohara, Hisahiro Matsubara

**Affiliations:** 1 Department of Frontier Surgery, Graduate School of Medicine, Chiba University, Chiba, Japan; 2 Anaut Inc, Tokyo, Japan; 3 Department of Gastroenterological Surgery, Toranomon Hospital, Tokyo, Japan; 4 Department of Surgery, Yokohama City University, Kanagawa, Japan; 5 Department of General Surgery, Kumagaya General Hospital, Saitama, Japan; 6 Department of Gastroenterological Surgery, Hyogo College of Medicine, Hyogo, Japan

**Keywords:** Artificial intelligence (AI), Rectal cancer surgery, Nerve-like structures, Surgical complications, Surgical education

## Abstract

**Background:**

Artificial intelligence (AI) has the potential to enhance surgical practice by predicting anatomical structures within the surgical field, thereby supporting surgeons' experiences and cognitive skills. Preserving and utilising nerves as critical guiding structures is paramount in rectal cancer surgery. Hence, we developed a deep learning model based on U-Net to automatically segment nerves.

**Methods:**

The model performance was evaluated using 60 randomly selected frames, and the Dice and Intersection over Union (IoU) scores were quantitatively assessed by comparing them with ground truth data. Additionally, a questionnaire was administered to five colorectal surgeons to gauge the extent of underdetection, overdetection, and the practical utility of the model in rectal cancer surgery. Furthermore, we conducted an educational assessment of non-colorectal surgeons, trainees, physicians, and medical students. We evaluated their ability to recognise nerves in mesorectal dissection scenes, scored them on a 12-point scale, and examined the score changes before and after exposure to the AI analysis videos.

**Results:**

The mean Dice and IoU scores for the 60 test frames were 0.442 (range 0.0465–0.639) and 0.292 (range 0.0238–0.469), respectively. The colorectal surgeons revealed an under-detection score of 0.80 (± 0.47), an over-detection score of 0.58 (± 0.41), and a usefulness evaluation score of 3.38 (± 0.43). The nerve recognition scores of non-colorectal surgeons, rotating residents, and medical students significantly improved by simply watching the AI nerve recognition videos for 1 min. Notably, medical students showed a more substantial increase in nerve recognition scores when exposed to AI nerve analysis videos than when exposed to traditional lectures on nerves.

**Conclusions:**

In laparoscopic and robot-assisted rectal cancer surgeries, the AI-based nerve recognition model achieved satisfactory recognition levels for expert surgeons and demonstrated effectiveness in educating junior surgeons and medical students on nerve recognition.

**Supplementary Information:**

The online version contains supplementary material available at 10.1007/s00464-024-10939-z.

According to a report on human performance errors during surgery, approximately 30% of all surgical complications are caused by misrecognition of anatomical structures [1]. Fatigue from long shifts and lengthy surgeries can reduce a surgeon's cognitive abilities and performance. Cognition and attention may also fluctuate depending on the surgeon's physical and mental states. Inexperienced surgeons also tend to lack knowledge and skills of anatomical structures to manage intraoperative events such as bleeding [2]. Technological innovations that support the surgeon's “cognition” can thus improve surgical outcomes.

In recent years, significant progress has been made in research on artificial intelligence (AI), and efforts are being made in various fields to apply AI to actual surgery. We previously reported the construction of a highly accurate recognition system for loose connective tissue fibres that defines a safe dissection plane in surgical videos using AI during lymphadenectomy with the help of intraoperative videos of robot-assisted gastrectomy [3]. The performance of this method was quantitatively demonstrated and qualitatively supported by experts. Notably, almost no misrecognitions occurred. This study is the first to demonstrate that AI developed through deep learning can precisely identify fine surgical anatomy. By training deep learning algorithms, fine and difficult-to-distinguish anatomical structures can be accurately predicted from intraoperative videos at a convincing level.

Our next step was to begin the segmentation of nerve fibres, which play an important role during surgery. Complications due to nerve damage remain a major challenge in rectal cancer surgery. Among patients undergoing surgery for stage II and III lower rectal cancer, dysuria or long-term urinary retention has been reported in 2.4% of cases, and sexual dysfunction has been reported in 14% [4]. Therefore, we developed a model for recognising nerves, including the hypogastric and pelvic splanchnic nerves, during rectal cancer surgery and evaluated its usefulness.

Additionally, we developed a neurocognitive AI model and examined its usefulness as an educational tool. Nerves are often perceived as indistinct and pale structures during surgery, and young surgeons are unable to recognise them during surgery. We examined whether it is possible for young surgeons to easily visualise nerves by watching a nerve recognition video analysed using AI, with the goal of making this system an educational tool for young surgeons to acquire the recognition skills of experienced surgeons as quickly as possible.

## Methods

### Video dataset

Surgical videos were obtained from Chiba University Hospital, Hyogo College of Medicine Hospital, and Toranomon Hospital after passing ethical reviews. Seventy surgeries performed between April 2019 and April 2023 were analysed, including robot-assisted rectal cancer operations performed using the da Vinci Xi and Xi + surgical system (Intuitive Surgical, Sunnyvale, CA, USA) and laparoscopic rectal cancer operations performed using VISERA ELITE II (Olympus, Tokyo, Japan). The recording systems (HVO-3300MT, SONY, Tokyo, Japan; AVCCAM AG-MDR15, Panasonic, Osaka, Japan) produced videos at 30 frames per second (fps). The videos were then categorised according to their use: 70 for training and validation of the algorithm and 5 for evaluation.

### Annotation and deep learning

We extracted scenes from surgical videos in which the nerve fibres of interest were present and annotated the structures that appeared to be nerve fibres. However, these annotations were image-based; therefore, definitive pathological confirmation was impossible. Therefore, we referred to the highlighted structures as nerve-like structures (NLSs). Scenes from the training videos in which NLSs were depicted were saved as still images in BMP format at a resolution of 1920 × 1080 pixels (aspect ratio 16:9). To create the training set, three surgeons (N.K., K.K., and T.M.) who had completed a fellowship in gastrointestinal surgery and an artist (M. Y.) who had been taught by these surgeons to recognise NLSs in surgery videos annotated the boundaries of each NLS in each frame.

The neural network model is based on the convolutional neural network (CNN) U-net architecture, which has shown promising results in segmentation tasks, particularly for medical images [5–7]. Our deep learning algorithm allowed for a more accurate output of segmentation maps by extracting object features in the convolution layer while restoring positional information in the deconvolution layer. The model training and inference were performed on a workstation with a Tesla V100 GPU (NVIDIA Corp., Santa Clara, CA, USA) and 32 GB of memory. Automated segmentation results are presented by highlighting the NLS area in yellow-green.

### Development of the AI model

A prototype AI model was developed using 666 images from the training data. The U-net deep learning algorithm was developed by augmenting the training data with annotations from surgeons. The development process from the initial prototype to the latest model involved more refined annotations and data augmentation without changing the U-Net architecture. The performance of the developed AI model was verified using validation videos separated from the training videos. The latest AI model was trained using 740 images captured from 70 video clips.

### The model evaluation by computation

**Fig. 1:**
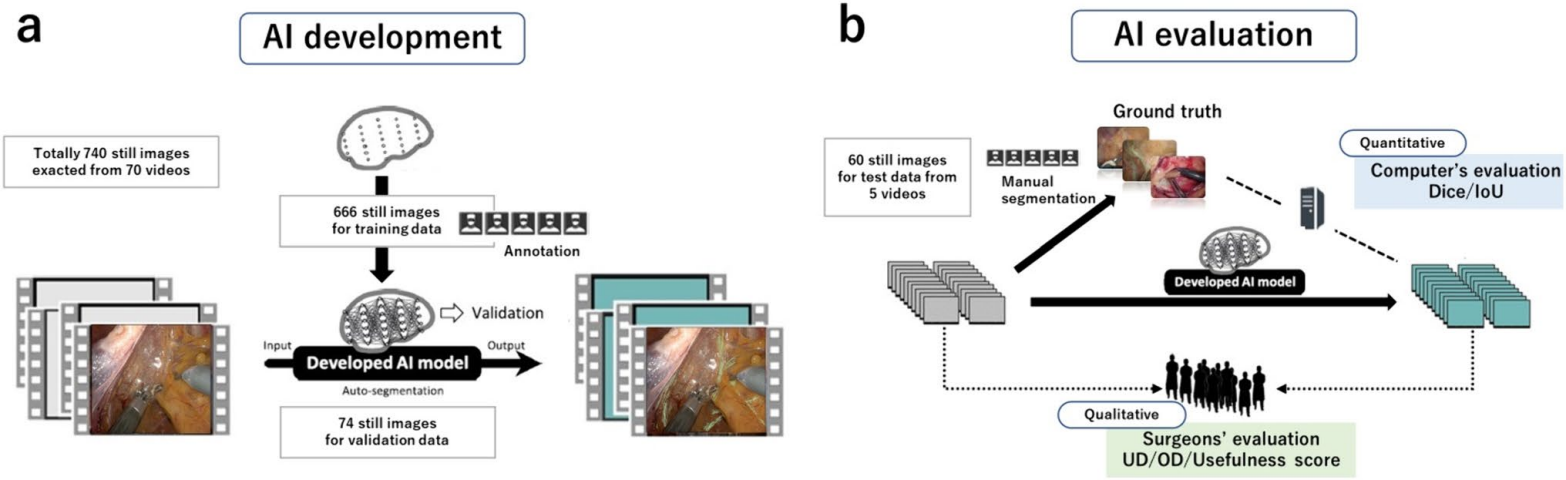
**a** The flowchart of the AI development method. The method uses 740 still images extracted from 70 videos of surgical procedures. The images are divided into training data and validation data. The training data consists of 666 still images that are annotated with black rectangles and white dots to mark the regions of interest. The validation data consists of 74 still images that are used to test the performance of the AI model. **b** The flowchart of the AI evaluation method. The method consists of two parts: quantitative and qualitative. The quantitative evaluation was performed by computerized Dice/IoU score. The qualitative evaluation was performed by expert surgeons using the UD/OD/Usefulness score

Our engineers randomly sampled 60 frames from five evaluation videos that underwent NLS prediction using the latest AI model (Fig. 1a). Five annotators manually segmented the corresponding frames from the original ones to obtain the ground truth. Quantitative evaluation of the agreement involved measuring the spatial overlap of pixels between the area manually segmented by the surgeons (considered the ground truth) and the area predicted from the AI's automated segmentation. To evaluate sensitivity and similarity, this assessment employed Dice [8, 9], Intersection over Union (IoU) [10], and scores, which are widely used performance metrics in machine learning. These metrics were calculated to measure the agreement between the predicted area of the automated segmentation of the AI and the actual area corresponding to the surgeons' manual segmentation as follows:

$$\begin{gathered} {\text{Dice }} = {\text{ TP }}/ \, \left( {{\text{TP }} + { 1}/{2 }\left( {{\text{FP }} + {\text{ FN}}} \right)} \right) \hfill \\ {\text{IoU }} = {\text{ TP }}/ \, \left( {{\text{TP }} + {\text{ FP }} + {\text{ FN}}} \right) \hfill \\ \end{gathered}$$

where TP, FN, and FP represent the true-positive, false-negative, and false-positive counts, respectively.

### The model evaluation by trained surgeons

Interpreting the values of quantitative evaluations such as Dice and IoU scores can be challenging for clinicians when assessing their clinical applicability, particularly in evaluations involving visual or cognitive performance. Therefore, we created a questionnaire to complement the quantitative evaluation.

**Fig. 2:**
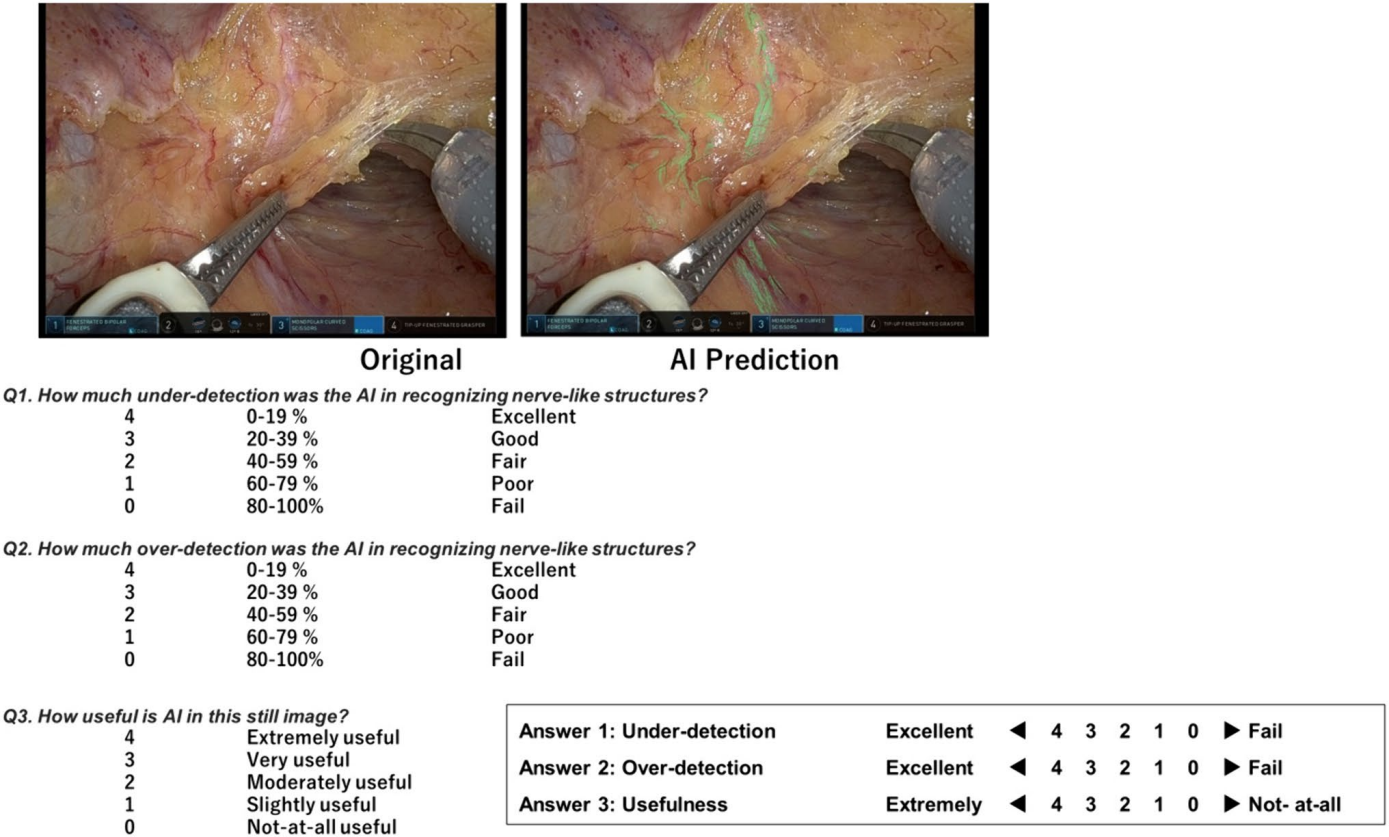
The questionnaire for qualitative evaluation of the AI’s segmentation performance completed by expert surgeons. The degrees of under-detection, over-detection, and usefulness were scored on a 5-point scale

We selected 60 frames from the evaluation videos that depicted a relatively high number of NLSs and conducted a qualitative evaluation using a 3-item questionnaire administered by five trained colorectal surgeons (Fig. 1b). Each test frame was sequentially projected onto a high-resolution screen alongside the original frame (Fig. 2), and the evaluators answered the questionnaire intuitively. The first question was *Q1. What was the degree of under-detection for the AI in recognising nerve-like structures?* The answers were scored on a 5-point scale with a 20% increment (4 for the lowest underdetection [0%–19%] to 0 for the highest underdetection [80%–100%]). The mean score of each frame was used as the under-detection (UD) score. The second question was *Q2. What was the degree of over-detection for the AI in recognising nerve-like structures?* The answers were scored on a 5-point scale with a 20% increment (4 for low overdetection [0–19%] to 0 for highest overdetection [80–100%]). The mean score of each frame was used as the over-detection (OD) score. The third question was *Q3. How useful is AI in this still image?* The answers were scored on a 5-point scale (0 = lowest not-at-all useful to 4 = highest extremely useful). The mean score of each frame was used as the usefulness score.

### Verification of educational effectiveness by AI recognition video

First, we assessed the recognition status of pelvic nerves among gastrointestinal surgeons. We conducted the study with 23 gastrointestinal surgeons from our department, including 4 colorectal specialists, 6 non-colorectal specialists, 11 rotating residents with undecided specialties, and 2 trainee doctors. The median number of years of medical experience among the physicians was 12 (range, 3–27 years).

**Fig. 3:**
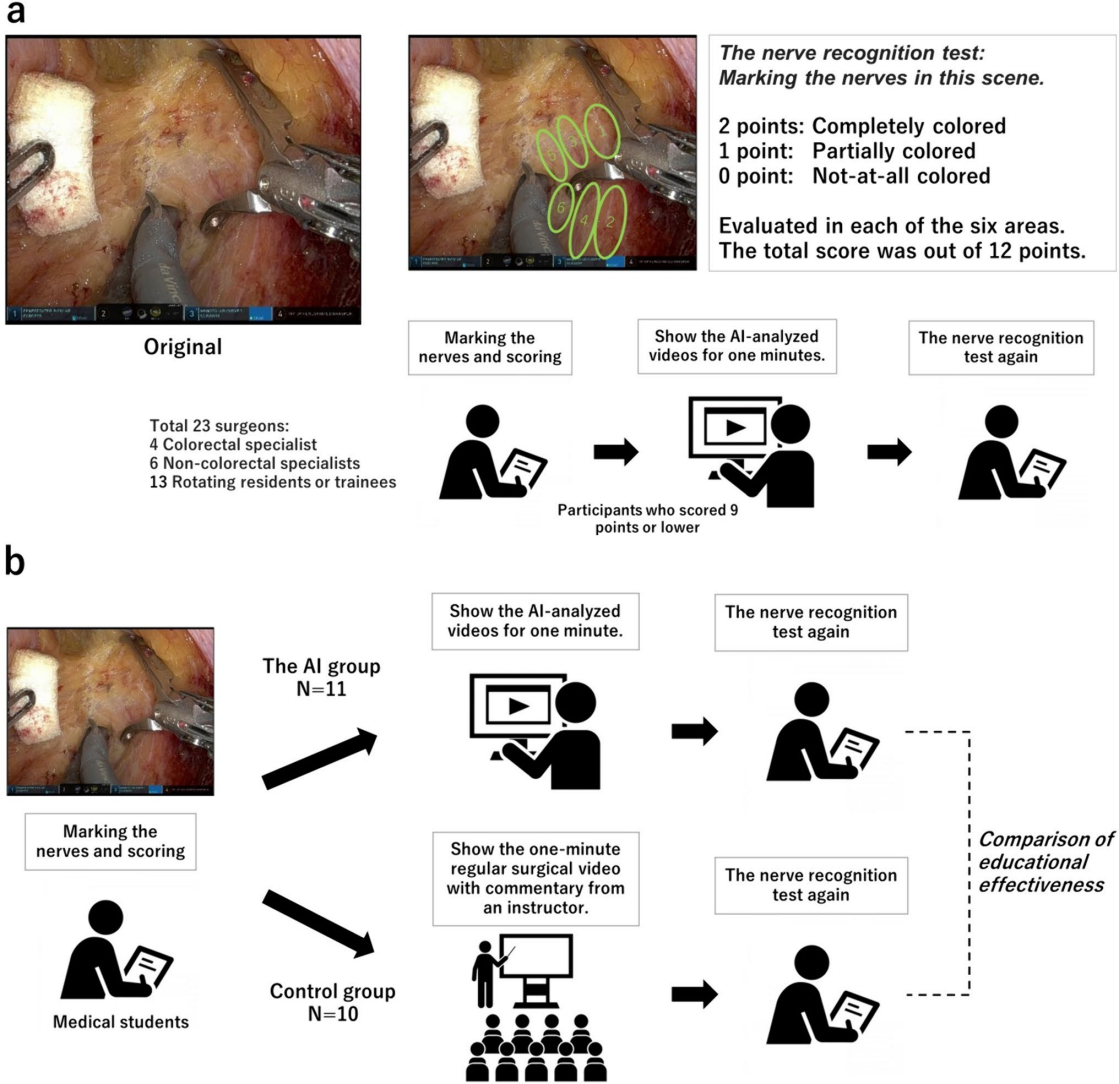
**a** The methods of evaluating the educational effectiveness of the AI nerve recognition model: 22 surgeons in our department were tested on how well they could color the nerves in this right rectal mesentery scene. Those who scored ≤ 9 points out of 12 were shown a 1-min AI nerve recognition video of a different case and scene and then retested to observe the change in their scores. **b** The educational effect of the AI nerve recognition model on medical students: This was a test conducted with 21 fifth-year medical students. The educational impact was compared between the group (n = 11) that watched AI nerve recognition videos and the group (n = 10) that received surgical video lectures with explanations from colorectal specialists

Participants were asked to mark the NLSs recognised by expert colorectal surgeons in six regions on the right side of the mesorectal fascia during the dissection scene (Fig. 3a). A scoring system was used in which participants received 2 points if they accurately marked all regions, 1 point if they partially marked some regions, 0 points if they did not mark any regions, -1 point if they marked outside the designated region once, and -2 points if they marked outside the designated region more than once. The mean total score was 12. The participants who scored ≤ 9 points in the nerve recognition evaluation were shown an AI-analysed video of a mesorectal dissection scene on the left side of the rectal mesentery, featuring a different patient, for 1 min. After viewing the video, the participants underwent another nerve recognition test. The scores were compared before and after viewing the AI-assisted nerve recognition videos.

We further validated the educational effectiveness of the AI video for medical students who completed their clinical rotations in our department (Fig. 3b). The students were classified into two groups: the AI group (11 students), who watched the AI video for 1 min without any commentary from the instructor, and the control group (10 students), who watched a 1-min regular surgical video with commentary from the instructor. They were asked to annotate the nerves in the mesorectal dissection scene on the right side before and after watching the videos using the scoring system mentioned earlier. We examined whether there was a difference in the rate of score improvement between the two groups.

### Statistical analyses

The UD and OD scores were plotted as scatter diagrams and confidence ellipses, respectively, with a probability of 0.95. The JMP Pro version 15 software program (SAS Institute Inc., Cary, NC, USA) was used for the statistical analyses.

## Results

**Fig. 4:**
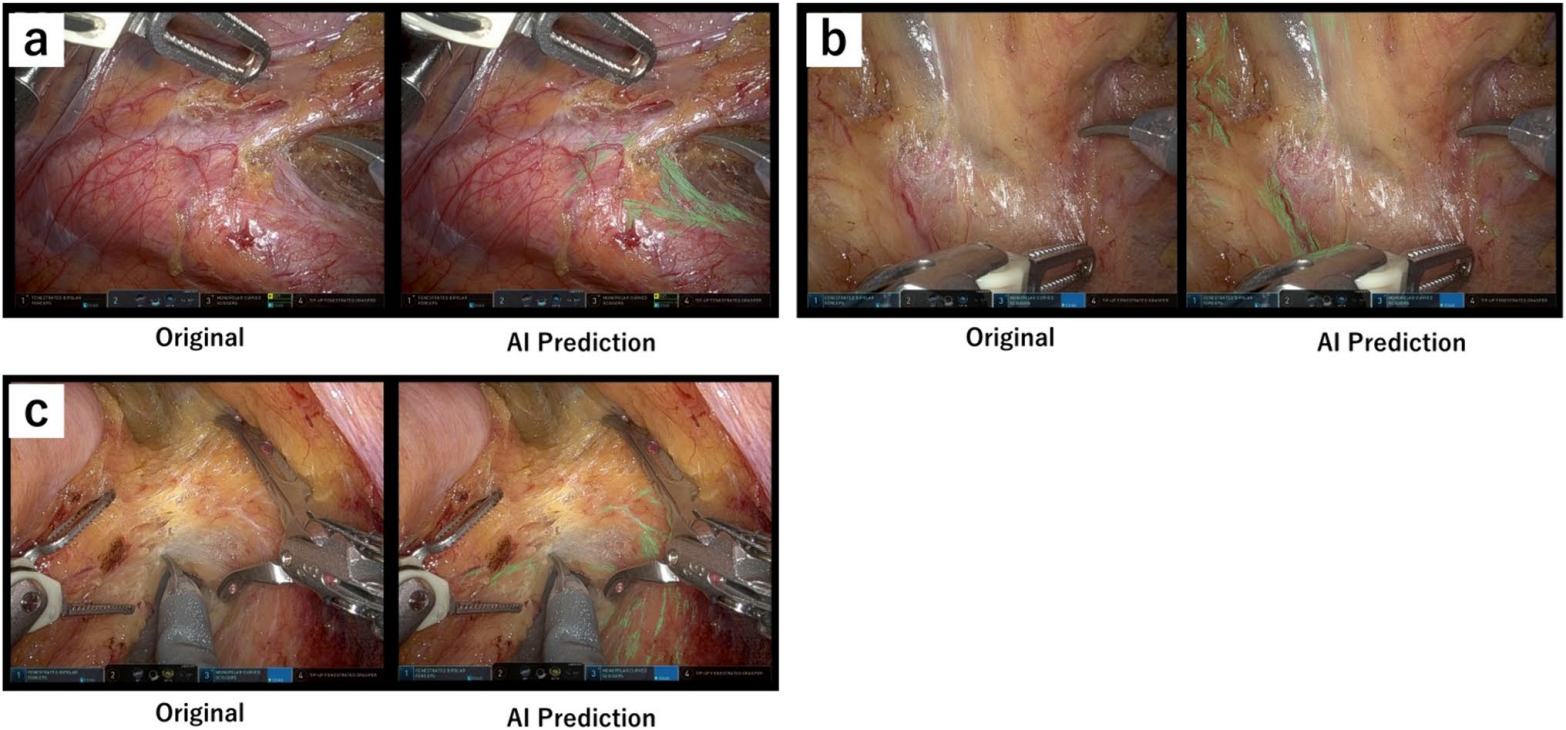
Examples of nerve recognition scenes by AI in rectal cancer surgery: Superior hypogastric plexus (**a**), hypogastric nerves (**b**), and pelvic visceral plexus (**c**). The right and left sides are the original and predicted AI, respectively

Figure 4 shows the outcomes of automated segmentation using the AI neural recognition model for several rectal cancer surgery scenarios. AI models have already acquired the ability \to roughly emphasise features related to the NLSs by analysing a large number of image pixels depicting different anatomical structures (such as arteries, lymph nodes, and fat tissue) and surgical instruments. The Electronic Supplementary Material (Video 1) provides illustrations that compare the outputs generated by the latest AI model with those of the original model.

In the depiction of the superior hypogastric plexus from the inferior mesenteric artery to the aortic bifurcation and the course of the hypogastric nerves around the sacral promontory, as well as during mesorectal dissection, the pelvic visceral nerve plexuses are highlighted in light green. The fibres that are emphasised in light green in these areas could not be definitively classified as nerve fibres based on pathological examination. Therefore, we designated these NLSs.

The mean Dice and IoU scores of the 60 test frames were 0.442 (range 0.0465–0.639) and 0.292 (range 0.0238–0.469), respectively, indicating acceptable sensitivity and similarity between the automated and manual segmentations. These 60 test frames were used for the qualitative evaluation. Supplemental Table 1 summarises the performance metrics measured by computation and the qualitative scores assigned by the evaluators for each of the 60 frames.

**Fig. 5:**
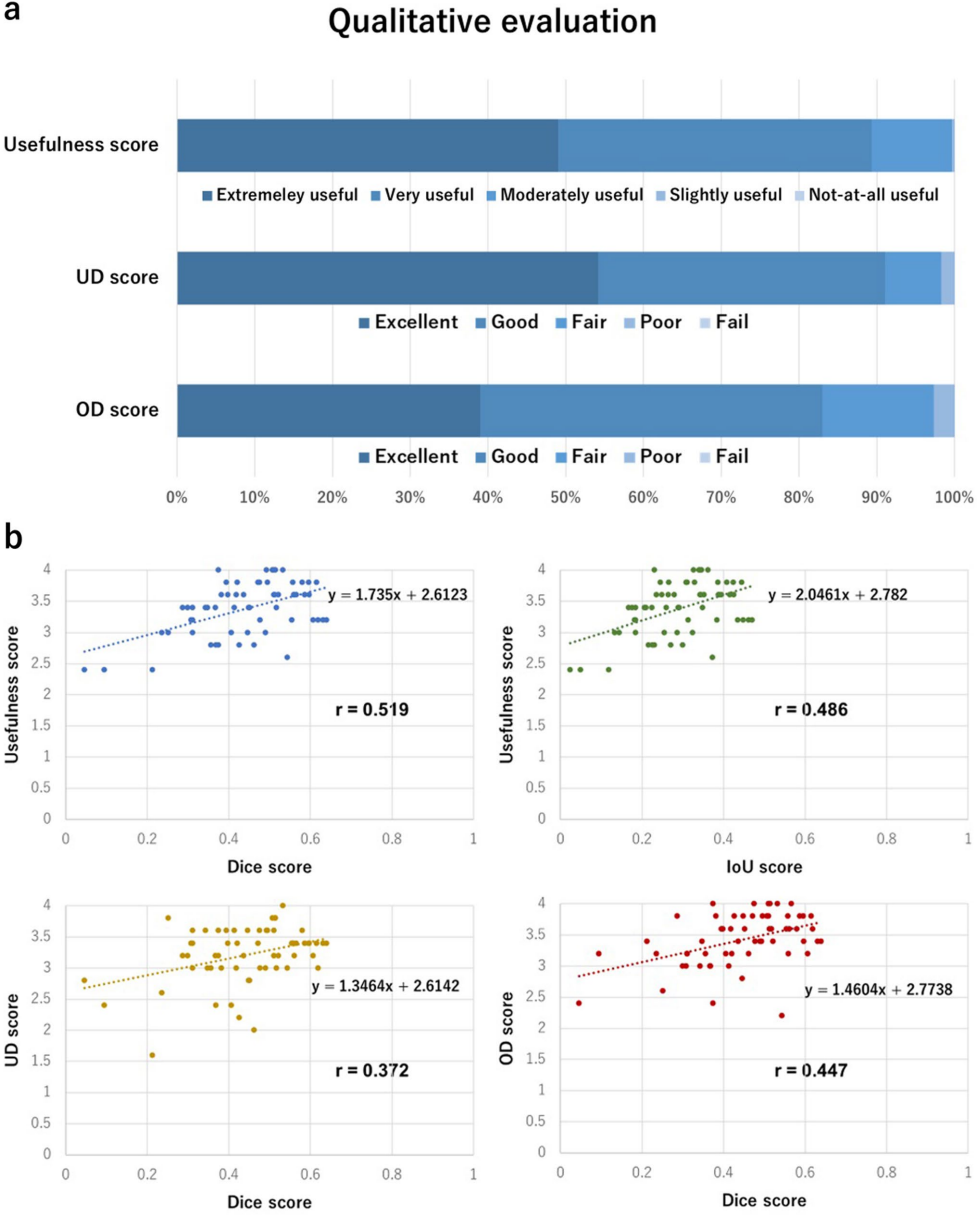
Results of the qualitative evaluation and the relationship between the qualitative and quantitative evaluation. **a** Results of the quantitative evaluation by five colorectal surgeons. **b** Relationships between computed performance metrics and qualitative scores. The scatter plot shows the relationships between the usefulness score and Dice/IoU score as well as among the UD, OD, and Dice scores. The correlation coefficient between the usefulness score and Dice score was r = 0.519 (blue scatter plot), and the correlation coefficient between the usefulness score and IoU score was r = 0.486 (green scatter plot). The correlation coefficient between the UD and Dice scores was r = 0.372 (yellow scatter plot), and the correlation coefficient between the OD and Dice scores was r = 0.447 (red scatter plot) (Color figure online)

In the qualitative evaluation by surgeons, the mean UD score was 3.21 (± 0.46), the mean OD score was 3.42 (± 0.41), and the mean usefulness score was 3.38 (± 0.43). Of all frames, 83.3% had a UD score of ≤ 1, 91.6% had an OD score of ≤ 1, and 85.0% had a usefulness score of ≥ 3. This result indicates that the evaluators were generally satisfied with the HLS segmentation output of the AI. Figure 5a shows the scores as percentages on a bar graph for the three qualitative evaluation questions.

The scatter plot in Fig. 5b illustrates the relationship between the usefulness score and the Dice/IoU score, as well as the relationships between the UD, OD, and Dice scores. The correlation coefficient (r) between the usefulness and dice scores was 0.519, and that between the usefulness and IoU scores was 0.486. Furthermore, the r between the UD and Dice scores was 0.372, and that between the OD and Dice scores was 0.447. These relationships were relatively weak, and there was some divergence between the qualitative and quantitative results.

**Fig. 6:**
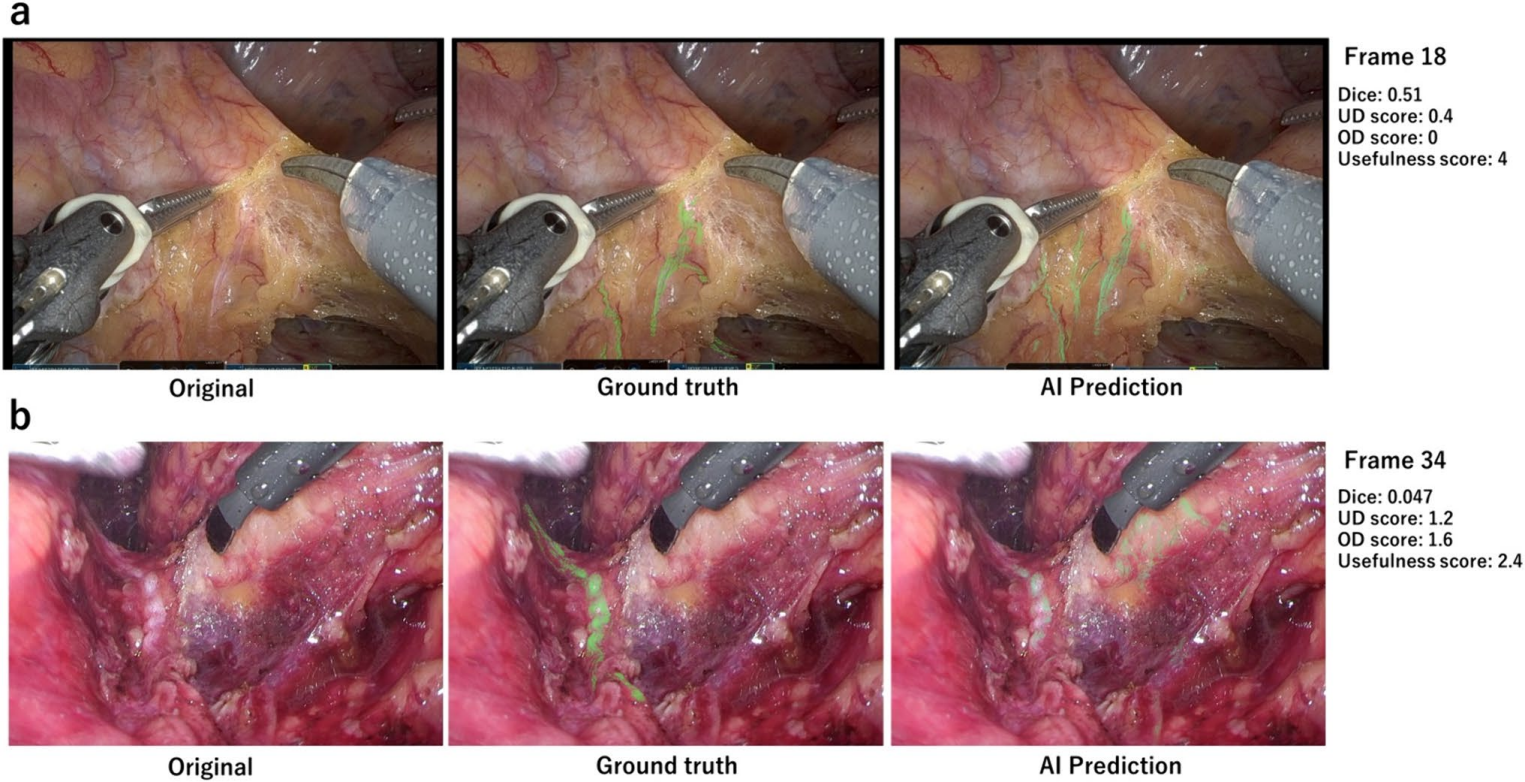
AI prediction results for (**a**) frame 18 with one of the highest Usefulness scores and (**b**) frame 34 with one of the lowest Usefulness scores. The yellow arrows indicate areas of over-detection and under-detection (Color figure online)

Figure 6 shows two examples of AI predictions. In Frame 18 (Fig. 6a), the AI segmentation of the NLSs deviated slightly from the manually segmented areas of the ground truth. The UD/OD score assigned by the surgeons was 0.4/0.0. This frame received the highest score for the usefulness of all surgeons. However, the Dice score was only 0.51, which was not particularly high. This may be due to the AI overemphasising slight deviations.

In frame 34 (Fig. 6b), there is a clear discrepancy between the AI segmentation results and ground truth. Surgeon evaluations were the lowest for this frame, with a UD/OD score of 1.2/1.6 and a usefulness score of 2.4. The Dice score was the lowest at 0.047. This was attributed to the difficulty in segmenting the nerve fibres because of the surrounding redness caused by blood.

**Fig. 7:**
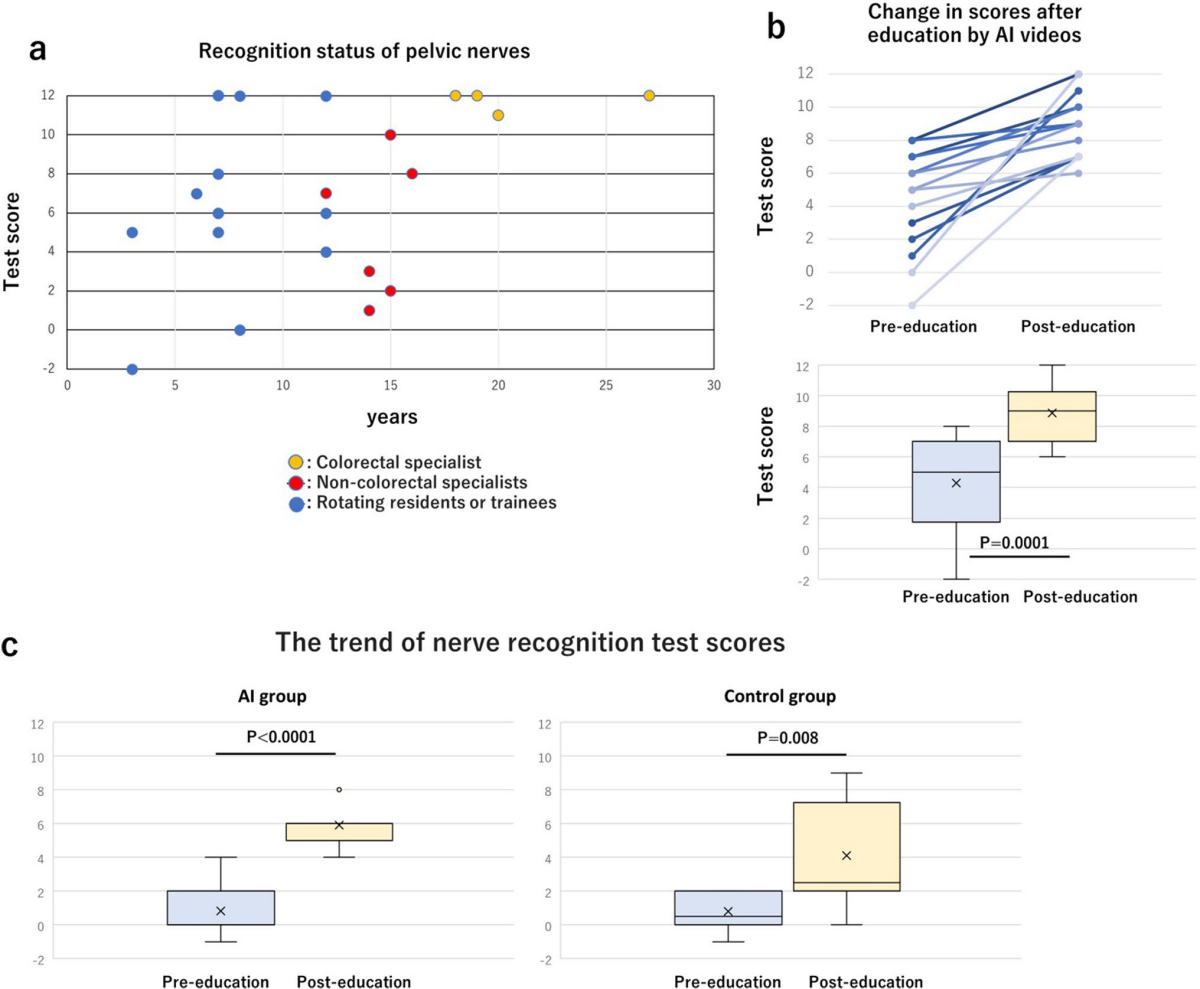
Results of nerve recognition test scores by proficiency level (**a**) and changes in nerve recognition test scores after watching the AI nerve recognition videos (**b**). **c** Changes in nerve recognition test scores among medical students. This table shows the mean and standard deviation of test scores for the AI group and the control group (Color figure online)

We also evaluated the educational effects of the AI nerve recognition videos. The status of pelvic nerve recognition by the surgeons is shown in Fig. 7a. In terms of recognising the NLSs, colorectal specialists achieved an average score of 11.75 (± 0.43). In contrast, non-colorectal specialists had an average score of 5.17 (± 3.33) points, and rotating residents with undecided specialities or trainees had an average score of 6.25 (± 4.26) points. Participants who scored ≤ 9 points were shown a 1-min AI nerve recognition video of a different patient and scene. The subsequent recognition of NLSs was assessed, and the results are shown in Fig. 7b. The average score pre-education was 4.28 (± 2.99) points, but post-education, it increased to 8.85 (± 1.88) points.

Verification results of the educational effects of nerve recognition on medical students are also presented. The medical students’ background characteristics are presented in Supplemental Table 2. There were no marked differences in the background characteristics between the AI group (N = 11) and the control group (N = 10). The AI group had a 5.1-point increase in their mean test score after watching the AI videos, which was 1.8 points higher than that of the control group. Furthermore, the AI group showed a 2.3-point decrease in the standard deviation of the test score after watching the AI videos, which was 1.4 points lower than that of the control group (Fig. 7c). Test score growth was also higher in the AI group than in the control group, with less variability.

## Discussion

AI algorithms, especially those related to deep learning, have made significant advancements in medical image recognition tasks such as X-ray imaging [11–13], endoscopy [14, 15], and pathological diagnoses [16, 17].

Some actively researched AI topics in the field of surgery include identifying surgical instruments and recognising surgical procedures such as cholecystectomy [18–20], colon resection [21], and sleeve gastrectomy [22]. The present study demonstrated the feasibility of using AI to automatically segment NLSs in intraoperative videos of laparoscopic and robot-assisted rectal cancer surgeries, highlighting the nerve pathways in each scene. The performance of the method was quantitatively evaluated, with a mean Dice score of 0.442 and a mean IoU score of 0.292, which were qualitatively assessed by expert colorectal surgeons, showing the high recognition capability of AI for nerves.

The validity of the Dice and IoU scores merits further discussion. Madani et al. reported high IoU scores for identifying the liver, gallbladder, and hepatocystic triangle in laparoscopic cholecystectomy: 0.86 (± 0.12), 0.72 (± 0.19), and 0.65 (± 0.22), respectively [18]. Mascagni et al. reported an average IoU of 0.66 for the segmentation of the critical view of safety in laparoscopic cholecystectomy [23]. In the present study, the mean Dice and IoU scores of 0.442 and 0.292, respectively, were not very high. However, as shown in Supplementary Video (Video 1), the AI clearly highlights the NLSs without any visual disagreement. Indeed, these subjective impressions are supported by the results shown in Fig. 5, as most surgeons were convinced by the AI’s prediction of NLSs. Anatomical structures such as the liver and gallbladder are often defined relatively clearly based on their shape and structural consistency, leading to improved accuracy in the segmentation results. However, the ambiguity and diversity of boundaries can pose challenges and increase the difficulty of segmenting more complex tissues or shapes, such as nerve fibres. These factors suggest that the results may vary depending on the object being segmented and that the adoption of metric-sensitive loss functions is important [9].

In our previous study, the Dice score for the segmentation of loose connective tissues using AI was 0.525 (range: 0.263–0.712), which was higher than that obtained for the segmentation of NLSs in this study [1]. In still images extracted from surgical videos, nerve fibres tend to appear more ambiguous than loose connective tissues, making annotation extremely challenging and prone to inconsistencies.

The people who performed the surgery and annotation in the previous study were not the same as those in the present study. When creating the ground truth or annotation for training data, we discuss where to annotate with expert surgeons to make the annotations as objective as possible. However, it is impossible to prove histologically that the annotated area is truly a nerve, which is why this AI model was not 100% correct. However, this AI model can still be very useful because, during surgery, surgeons must independently determine whether a structure is a nerve, often without the opportunity for discussion, within a limited timeframe.

Furthermore, the most crucial aspect of its utility as an intraoperative navigation system is whether this AI model can perform real-time analysis during surgery, which is an issue that has already been resolved. The analysis speed of this AI model has already reached 60fps, making real-time analysis feasible in the operating room.

Additionally, we verified the educational effects of the AI nerve recognition model. As shown in Fig. 7a, when examining the test scores for pelvic nerve recognition, it becomes evident that there is considerable variation in nerve recognition proficiency among surgeons and residents who do not specialise in colorectal surgery. As shown in Fig. 7b, when comparing the results before and after watching the AI nerve recognition model analysis video, all participants with test scores ≤ 8 points achieved an increase in their test scores. The standard deviation decreased from 2.98 to 1.88.

In a similar test conducted with medical students, the educational effectiveness of the AI nerve recognition model was compared with that of a lecture by a colorectal surgeon. The AI nerve recognition model involved viewing AI-analysed videos for only 1 min, whereas a lecture on pelvic nerve recognition by colorectal surgeons used actual surgical videos for approximately 15 min. The AI nerve recognition model accurately identified fine anatomical landmarks, such as neural structures and detachable layers, in surgical videos and presented the results as image segmentations. This enabled surgeons to learn the key points and nuances of nerve recognition visually. Verbal lectures often struggle to describe intricate fibrous structures that are difficult to explain using words or diagrams [24, 25].

Our results suggest the model’s potential for clinical applications; however, there are several limitations that must be considered. The accuracy of the AI model in recognising nerves can be compromised under complex intraoperative conditions such as bleeding. Overcoming this issue is critical for achieving our goal of enhancing safety by combining surgery and AI technology. We believe that focusing on collecting scenes with lower accuracy, such as bleeding scenes, from a larger set of surgical videos for training will help improve the segmentation performance. Additionally, AI models are still unapproved medical software programs, and this qualitative evaluation was not based on qualitative assessments conducted by surgeons during actual surgery. Further safety validation and regulatory approval are required, along with qualitative assessments by surgeons in clinical settings. Furthermore, the versatility of this method must be evaluated. AI models make inferences and predictions based on training datasets. NLSs are common anatomical features in many surgical areas. We previously confirmed that an algorithm trained on rectal resection datasets can recognise NLSs with significant accuracy in gastrectomy videos. The recognition of the vagus nerve in thoracic surgery areas has also been confirmed.

These considerations highlight the potential challenges that arise when AI is integrated into surgical practice. As technology continues to evolve, addressing these limitations is essential to ensure safe and accurate surgical procedures.

## Conclusions

In laparoscopic and robot-assisted rectal cancer surgery, the AI nerve recognition model recognises NLSs at a level that is attractive to colorectal specialists, such as identifying intricate and hard-to-discern NLSs in intraoperative videos. Furthermore, viewing the recognition videos generated by the AI model had significant educational benefits. This technology not only aids in real-time decision-making during surgery, thereby reducing adverse events caused by nerve damage, but also serves as an educational tool for young surgeons. As a future perspective, we aim to examine in a multi-center collaborative study whether this AI technology reduces clinical complications and to evaluate its educational impact in terms of anatomical recognition.

### Supplementary Information

Below is the link to the electronic supplementary material.

Supplementary file1 (DOCX 26 KB)

Supplementary file2 (MP4 299787 KB)

## Data Availability

This study was approved by the Ethics Committee of Graduate School of Medicine, Chiba University, Chiba, Japan. Informed consent was obtained from all participants for research purposes. We cannot share data and materials because the Ethics Committee of the Graduate School of Medicine, Chiba University, prohibits the publication of a raw database that includes patients’ clinical data, even in cases where identification/confidential data are not included.
