# Correction: An artificial intelligence-based nerve recognition model is useful as surgical support technology and as an educational tool in laparoscopic and robot-assisted rectal cancer surgery

**DOI:** 10.1007/s00464-024-11132-y

**Published:** 2024-08-12

**Authors:** Kazuya Kinoshita, Tetsuro Maruyama, Nao Kobayashi, Shunsuke Imanishi, Michihiro Maruyama, Gaku Ohira, Satoshi Endo, Toru Tochigi, Mayuko Kinoshita, Yudai Fukui, Yuta Kumauzu, Junji Kita, Hisashi Shinohara, Hisahiro Matsubara

**Affiliations:** 1 Department of Frontier Surgery, Graduate School of Medicine, Chiba University, Chiba, Japan; 2 Anaut Inc., Tokyo, Japan; 3 Department of Gastroenterological Surgery, Toranomon Hospital, Tokyo, Japan; 4 Department of Surgery, Yokohama City University, Kanagawa, Japan; 5 Department of General Surgery, Kumagaya General Hospital, Saitama, Japan; 6 Department of Gastroenterological Surgery, Hyogo College of Medicine, Hyogo, Japan

**Correction: Surgical Endoscopy** 10.1007/s00464-024-10939-z

The original online version of this article was revised to correct the spelling of coauthor Yuta Kumazu. The affiliation of coauthors Yuta Kumazu and Nao Kobayashi was also corrected to Anaut Inc., Tokyo, Japan.

The original article has been corrected.

