# Supplementary material for: An artificial intelligence-based nerve recognition model is useful as surgical support technology and as an educational tool in laparoscopic and robot-assisted rectal cancer surgery
Source: Surg Endosc. 2024 Jul 29;38(9):5394–404. doi: 10.1007/s00464-024-10939-z (PMC11362368; doi:10.1007/s00464-024-10939-z)
Supplement: Supplementary file 1 — Supplementary file1 (DOCX 26 KB) [file 464_2024_10939_MOESM1_ESM.docx]

**Supplemental Table. 1**

| **Frame** | **Dice** | **IoU** | **UD** | **OD** | **Usefulness** |
| --- | --- | --- | --- | --- | --- |
| 1 | 0.21 | 0.12 | 1.6 | 3.4 | 2.4 |
| 2 | 0.37 | 0.23 | 2.4 | 3 | 2.8 |
| 3 | 0.57 | 0.39 | 3.4 | 4 | 3.6 |
| 4 | 0.31 | 0.18 | 3 | 3.2 | 3 |
| 5 | 0.45 | 0.29 | 2.8 | 3.6 | 3.4 |
| 6 | 0.48 | 0.31 | 3 | 3.4 | 3.2 |
| 7 | 0.24 | 0.13 | 2.6 | 3.2 | 3 |
| 8 | 0.46 | 0.30 | 2 | 3.2 | 2.8 |
| 9 | 0.35 | 0.21 | 3 | 3.4 | 3.4 |
| 10 | 0.37 | 0.23 | 3.2 | 2.4 | 2.8 |
| 11 | 0.42 | 0.27 | 3.4 | 3.2 | 3.8 |
| 12 | 0.64 | 0.47 | 3.4 | 3.4 | 3.2 |
| 13 | 0.63 | 0.46 | 3.4 | 3.4 | 3.2 |
| 14 | 0.42 | 0.26 | 3 | 3.6 | 3.6 |
| 15 | 0.37 | 0.23 | 3.6 | 4 | 4 |
| 16 | 0.50 | 0.33 | 3.6 | 3.8 | 3.8 |
| 17 | 0.47 | 0.31 | 3.6 | 4 | 3.8 |
| 18 | 0.51 | 0.34 | 3.6 | 4 | 4 |
| 19 | 0.56 | 0.39 | 3.4 | 3.2 | 3.6 |
| 20 | 0.56 | 0.39 | 3.2 | 3.6 | 3.6 |
| 21 | 0.51 | 0.34 | 3.8 | 3.8 | 4 |
| 22 | 0.47 | 0.31 | 3.4 | 3.8 | 3.8 |
| 23 | 0.45 | 0.29 | 3.6 | 2.8 | 3 |
| 24 | 0.61 | 0.44 | 3.2 | 3.2 | 3.2 |
| 25 | 0.30 | 0.18 | 3.2 | 3 | 3.4 |
| 26 | 0.43 | 0.27 | 2.2 | 3.8 | 2.8 |
| 27 | 0.39 | 0.25 | 3.6 | 3.6 | 3.8 |
| 28 | 0.52 | 0.35 | 3.2 | 3.4 | 3.6 |
| 29 | 0.52 | 0.35 | 3 | 3.6 | 3.4 |
| 30 | 0.60 | 0.42 | 3.4 | 3.8 | 3.8 |
| 31 | 0.62 | 0.44 | 3.4 | 3.8 | 3.8 |
| 32 | 0.60 | 0.43 | 3.4 | 3.4 | 3.6 |
| 33 | 0.51 | 0.35 | 3.8 | 4 | 4 |
| 34 | 0.05 | 0.02 | 2.8 | 2.4 | 2.4 |
| 35 | 0.54 | 0.37 | 3 | 2.2 | 2.6 |
| 36 | 0.45 | 0.29 | 2.8 | 3.8 | 3.4 |
| 37 | 0.31 | 0.18 | 3.6 | 3.2 | 3.4 |
| 38 | 0.41 | 0.26 | 2.4 | 3.2 | 3 |
| 39 | 0.41 | 0.26 | 3.6 | 3 | 3.4 |
| 40 | 0.40 | 0.25 | 3.4 | 3.6 | 3.6 |
| 41 | 0.37 | 0.23 | 3.2 | 3 | 3.4 |
| 42 | 0.59 | 0.42 | 3.4 | 3.8 | 3.6 |
| 43 | 0.51 | 0.34 | 3.2 | 3.8 | 3.6 |
| 44 | 0.51 | 0.34 | 3.8 | 3.6 | 3.6 |
| 45 | 0.34 | 0.21 | 3.6 | 3 | 3.4 |
| 46 | 0.31 | 0.18 | 3.4 | 3 | 3.2 |
| 47 | 0.10 | 0.05 | 2.4 | 3.2 | 2.4 |
| 48 | 0.36 | 0.22 | 3 | 3.2 | 2.8 |
| 49 | 0.55 | 0.38 | 3.4 | 3.6 | 3.2 |
| 50 | 0.31 | 0.18 | 3.4 | 3.2 | 3.2 |
| 51 | 0.29 | 0.17 | 3.2 | 3.8 | 3.4 |
| 52 | 0.38 | 0.24 | 3 | 3.8 | 3.6 |
| 53 | 0.62 | 0.45 | 3 | 3.6 | 3.2 |
| 54 | 0.44 | 0.28 | 3.2 | 3.4 | 3.6 |
| 55 | 0.56 | 0.39 | 3.4 | 3.8 | 3.8 |
| 56 | 0.53 | 0.36 | 4 | 4 | 4 |
| 57 | 0.25 | 0.14 | 3.8 | 2.6 | 3 |
| 58 | 0.49 | 0.33 | 3.6 | 3.4 | 4 |
| 59 | 0.58 | 0.41 | 3.6 | 3.6 | 3.8 |
| 60 | 0.49 | 0.32 | 3 | 3.4 | 3 |
| **Mean** |  |  | 3.210 | 3.420 | 3.38 |
| **SD** |  |  | 0.458 | 0.414 | 0.424 |

**Supplemental Table. 2**

|  | **AI group**  **(N=11)** | **Control group**  **(N=10)** | ***p*-value** |
| --- | --- | --- | --- |
| **Age** | 22.5±0.8 | 22.9±0.6 | 0.26 |
| **Sex (male : female)** | 3 : 6 | 5 : 5 | 0.11 |
| **One's field of interest**  **(Surgery : Internal medicine : Undecided)** | 2 : 4: 5 | 2 : 4 : 4 |  |
| **Number of surgical observation** |  |  |  |
| **Total** | 9 (5-17) | 9 (1-12) | 0.17 |
| **Number of observation of rectal cancer surgery** | 0 (0-1) | 0 (0-1) | 0.92 |
